# Timing of DOAC Initiation After Atrial Fibrillation-Related Acute Ischemic Stroke: A Unified Protocol Harmonizing Clinical Safety and Imaging-Based Precision in the Post-CATALYST Era

**DOI:** 10.7759/cureus.105563

**Published:** 2026-03-20

**Authors:** Deb Mojumder, Apurva Popat

**Affiliations:** 1 Neurology, Marshfield Clinic Health System, Marshfield, USA; 2 Neurocritical Care, Ochsner Health, New Orleans, USA; 3 Cardiology, Marshfield Clinic Health System, Marshfield, USA

**Keywords:** anticoagulation timing, atrial fibrillation, catalyst, clinical protocol, direct oral anticoagulants, elan trial, hemorrhagic transformation, ischemic stroke, neuroimaging, secondary prevention

## Abstract

Atrial fibrillation-related ischemic strokes carry a high risk of early recurrence. Historically, anticoagulation was delayed by conservative *1-3-6-12 day* rules necessitated by the hemorrhagic risks of Vitamin K Antagonists. The 2025 CATALYST individual participant data meta-analysis and recent trials (ELAN, OPTIMAS, TIMING) have now established the safety of *ultra-early* direct oral anticoagulant initiation within four days. Yet clinicians still face the challenge of reconciling clinical severity with radiological findings, and critical distinctions between trial-level evidence and pooled meta-analytic inferences require explicit navigation.

This narrative review synthesizes landmark evidence into a practical framework that bridges high-level safety data and imaging-based precision. We present a unified seven-step clinical protocol for direct oral anticoagulant initiation that distinguishes evidence sources, prioritizing more conservative estimates when findings diverge. The protocol utilizes CATALYST safety criteria for screening while adopting ELAN's radiological infarct size tiers to dictate precise timing within a 0-4 day *Safe Zone *- interpreted as a target range, not a maximum threshold.

Key innovations include tiered hemorrhagic transformation management, validating early initiation in petechial infarction (HI1/HI2) while mandating delay for parenchymal hematomas (Days 6-7 for PH1; ≥4 weeks for PH2); integrated safety failsafe’s, including the *Conservative Discordance* rule to mitigate inter-reader variability and a categorical prohibition of antiplatelet bridging; and dynamic post-initiation monitoring emphasizing renal re-evaluation and an *anticoagulant-only* transition at discharge.

Synthesis confirms that direct oral anticoagulants offer superior safety over Vitamin K Antagonists, though a hierarchy of bleeding risk among agents informs drug selection without altering the timing window. The post-CATALYST era marks a definitive shift toward a streamlined, direct oral anticoagulant-first approach, though important uncertainties remain for patients with severe stroke, extremely large infarcts, extensive hemorrhagic transformation, pre-existing disability, or multiple exclusion risks-populations where evidence is insufficient and shared decision-making is essential.

This unified seven-step protocol provides an actionable framework for the multidisciplinary stroke team, replacing outdated Vitamin K Antagonist-era rules and hazardous antiplatelet bridging with individualized, imaging-directed care.

## Introduction and background

Acute ischemic stroke in patients with atrial fibrillation is a formidable clinical challenge, characterized by greater severity, higher mortality, and a disproportionately elevated risk of early recurrence compared to other stroke etiologies [1,2]. While effective anticoagulation is the cornerstone of secondary prevention, its initiation in the acute phase has long been fraught with the critical tension between preventing recurrent thromboembolism and precipitating hemorrhagic transformation of the index infarct.

For decades, this balance was dictated by the conservative *1-3-6-12 day* rule, a necessity of the Vitamin K Antagonist era. This framework, which delayed therapy based on clinical severity, was a direct response to the narrow therapeutic window and substantial risk of symptomatic intracranial hemorrhage associated with Vitamin K Antagonists [3,4]. The advent of Direct Oral Anticoagulants, with their superior safety profile, particularly a markedly lower risk of intracranial bleeding, fundamentally challenged this cautious paradigm [5,6]. Despite this, clinical inertia persisted, often manifesting as hazardous antiplatelet "bridging" -a practice now shown to offer no safety advantage, with a risk of intracranial hemorrhage that is statistically comparable to full-dose anticoagulation [7].

The publication of landmark randomized trials - ELAN, OPTIMAS, and TIMING - culminating in the 2025 CATALYST individual participant data meta-analysis, has irrevocably shifted the landscape [8-11]. CATALYST definitively established that *ultra-early* direct oral anticoagulant initiation (≤4 days) significantly reduces recurrent ischemic stroke with no increase in symptomatic intracranial hemorrhage (0.4% in both groups), ushering in the *post-CATALYST era* of informed early initiation [11]. However, this new era presents a critical translational challenge. The meta-analysis provides a powerful but binary *safety floor* for the 0-4 day window, while the ELAN trial demonstrated that optimal timing is not uniform, but rather varies by radiological infarct size, from 48 hours for minor strokes to day 6-7 for major infarcts [8]. A clinician relying solely on the CATALYST estimate might mistakenly apply a one-size-fits-all approach, potentially under-treating minor strokes or exposing patients with major infarcts to unnecessary risk.

Furthermore, current guidelines, while advocating for individualized timing, lack the operational specificity required for day-by-day decision-making, particularly regarding how to integrate infarct size and the grade of hemorrhagic transformation [12,13]. This narrative review directly addresses this implementation gap. We present a unified, seven-step clinical protocol that harmonizes the high-level safety evidence of the CATALYST meta-analysis with the imaging-directed precision of the ELAN trial. By prioritizing radiological infarct volume and a tiered management strategy for hemorrhagic transformation, this framework moves beyond arbitrary timelines to provide an actionable pathway. It incorporates key safety failsafes, including the *Conservative Discordance* rule for imaging ambiguity and a categorical prohibition of antiplatelet bridging, and offers dynamic guidance for post-initiation monitoring, translating the advances of the post-CATALYST era into a structured, evidence-based tool for the multidisciplinary stroke team.

## Review

Methodology

This review was conducted with a focus on transparency and reproducibility, adhering to the reporting principles outlined in the Preferred Reporting Items for Systematic Reviews and Meta-Analyses (PRISMA) 2020 statement for narrative and scoping reviews [14]. A comprehensive literature search was performed across PubMed, Scopus, and the Cochrane Central Register of Controlled Trials for articles published between January 2010 and January 2026. Search terms included "direct oral anticoagulant," "atrial fibrillation," "ischemic stroke," "timing," "early initiation," and "hemorrhagic transformation."

The search targeted high-level evidence, prioritizing large-scale randomized controlled trials that have shaped contemporary practice [8-10]. The scope was further expanded to include individual participant data meta-analyses, most notably the CATALYST study [11], as well as relevant prospective registries and international guideline statements [12,13].

Statistical framing

To ensure a robust methodological foundation, this review distinguishes between trial-level results and broader meta-analytic inferences - a distinction central to the translational gap identified in the Introduction. Trial-level data provide granular information about specific patient phenotypes, imaging protocols, and precision-timing estimates, while meta-analytic estimates offer broader safety floors across heterogeneous populations. When trial-level and meta-analytic findings diverge, we prioritize the more conservative estimate for safety recommendations.

A critical nuance informing this approach concerns the ELAN trial's statistical design. Rather than aiming to establish definitive superiority or non-inferiority, the trial's sample size was calculated based on the width of expected confidence intervals to provide precise estimates of treatment effect [8]. Consequently, the timing windows derived from ELAN should be interpreted as evidence-based estimates for safe practice - or *Safe Zones *- rather than definitive proof that one specific day is statistically superior to another.

A narrative synthesis approach was chosen over a formal systematic meta-analysis because the primary objective is to bridge the gap between broad statistical safety data and the practical, imaging-based protocols required at the bedside. Studies focusing on Vitamin K Antagonists or non-atrial fibrillation stroke etiologies were excluded unless they provided essential historical context for the evolution of the *1-3-6-12 day* rule [3]. This methodology ensures that the resulting unified protocol is grounded in the most recent trial data while remaining applicable to diverse clinical scenarios.

Synthesis of landmark evidence

Primary Efficacy and Event Rates

The CATALYST meta-analysis demonstrated a significant reduction in the primary composite outcome of recurrent ischemic stroke, symptomatic intracranial hemorrhage, or unclassified stroke within 30 days. The primary outcome occurred in 2.1% of the early initiation group compared to 3.0% in the later group. This benefit was driven by a reduction in recurrent ischemic stroke (1.7% early vs. 2.6% delayed), corresponding to an absolute risk difference of 0.9% and a number needed to treat of 111 [11].

CATALYST pooled data from TIMING, ELAN, OPTIMAS, and START (*n* = 5,441), comparing early initiation (≤4 days) versus later initiation (≥5 days). The START trial, representing approximately 5% of the pooled cohort, did not materially influence the primary effect estimates but provided important confirmatory data on MRI-based timing stratification; sensitivity analyses excluding START showed consistent results [11].

Individual trials offer insights into specific patient phenotypes that pooled estimates cannot provide. The OPTIMAS trial demonstrated that high-risk cohorts - including the elderly and those with chronic kidney disease (CKD, CrCl >15 mL/minute) - do not require specialized delays, as early initiation showed no excess bleeding in these subgroups [9]. The ELAN trial introduced a precision-timing model based on radiological infarct size (detailed below), with timing stratified by infarct volume rather than clinical severity alone [8].

Subgroup analyses from ELAN suggest that the absolute benefit of early initiation may be greater in patients with major stroke compared to those with minor or moderate stroke [15]. For the primary composite outcome at 30 days, the odds ratios were 0.89 (95% CI 0.38-2.10) for minor stroke, 0.80 (95% CI 0.35-1.74) for moderate stroke, and 0.52 (95% CI 0.21-1.18) for major stroke. However, the study was not powered for interaction testing, and event numbers were low, limiting definitive conclusions about differential treatment effects across infarct size categories.

It is important to clarify that the ELAN trial was powered for the composite primary outcome comparing early versus late initiation, not for comparisons between individual direct oral anticoagulant agents [8]. While recurrence rates did not differ significantly by agent in exploratory analyses, the study was not designed to detect such differences, and agent-specific comparisons should be interpreted as hypothesis-generating only.

The TIMING trial established the non-inferiority of early initiation in clinically stable patients free of baseline hemorrhagic transformation [10].

The imaging eligibility criteria differed substantially across trials. ELAN required baseline MRI or CT with central adjudication of infarct size; OPTIMAS allowed site-reported imaging without central review; TIMING utilized clinical diagnosis without mandated imaging criteria for randomization; and START required MRI for all participants [8-10,16]. These differences affect generalizability: trials with rigorous imaging requirements (ELAN, START) provide stronger evidence for imaging-based timing but enrolled more selected populations, whereas trials with pragmatic designs (OPTIMAS, TIMING) are more generalizable but provide less granular imaging data. The consistency of the safety signal across these heterogeneous approaches strengthens the overall conclusion while highlighting the need for standardized imaging protocols in future research.

Recognizing these methodological nuances in imaging protocols, outcome definitions, and patient exclusions is critical for applying meta-analytic safety floors to individual bedside decisions - a granularity that pooled analyses like CATALYST necessarily aggregate but cannot dissect [8-10].

Understanding these trial-level nuances provides context for appreciating the evolution of the anticoagulant therapies themselves, which we now review.

Evolution and Comparative Safety of Anticoagulant Therapies

Anticoagulant therapies for stroke prevention in atrial fibrillation have advanced markedly over decades, shifting from Vitamin K Antagonists like warfarin to direct oral anticoagulants, which provide improved efficacy, safety, and convenience [5,17,18]. Warfarin, introduced in the mid-20th century, served as the cornerstone of oral anticoagulation for over 50 years. Landmark trials in the late 1980s and early 1990s showed that warfarin reduced ischemic stroke risk by approximately 64% to 79% compared to placebo or antiplatelet therapy [19]. A meta-analysis confirmed that adjusted-dose warfarin reduces stroke by 64% compared to control [20]. In secondary prevention specifically, the European Atrial Fibrillation Trial demonstrated that anticoagulation reduces the annual risk of recurrent vascular events from 17% to 8%, preventing 90 vascular events per 1000 patients treated per year [21].

However, warfarin's limitations-including a narrow therapeutic window, frequent international normalized ratio monitoring, drug-food interactions, and variable dosing-restricted its optimal use [5,13]. The pivotal direct oral anticoagulant trials beginning in the late 2000s transformed practice. Dabigatran, the first direct oral anticoagulant approved for atrial fibrillation stroke prevention in 2010, demonstrated noninferiority to warfarin with lower intracranial hemorrhage risk [17]. Subsequent approvals included rivaroxaban (2011), apixaban (2012), and edoxaban (2015) [6,18,22]. These factor Xa inhibitors showed comparable or superior stroke prevention versus warfarin, with consistently lower intracranial hemorrhage rates and no need for routine monitoring [13,23].

It is important to acknowledge that during the initial direct oral anticoagulant approval era (2009-2013), all pivotal trials excluded patients with recent stroke (typically within 7-30 days), creating an evidence gap that persisted for nearly a decade. The timing trials (ELAN, OPTIMAS, TIMING) and CATALYST meta-analysis have now filled this gap, but the safety data for initiation within the first 48 hours derive almost exclusively from these recent studies, not the original registration trials.

The comparative safety of direct oral anticoagulants versus Vitamin K Antagonists in patients with recent acute ischemic stroke is a critical consideration. Systematic reviews and meta-analyses specific to atrial fibrillation patients with prior stroke indicate that direct oral anticoagulants offer a safer bleeding profile than Vitamin K Antagonists. A 2023 meta-analysis by Umashankar et al. found direct oral anticoagulants associated with significantly lower intracranial hemorrhage risk versus warfarin in non-claims/RCT data (HR 0.51, 95% CI 0.38-0.67), although this benefit was attenuated in claims-based studies [24]. Observational comparisons are subject to confounding by indication, as patients prescribed direct oral anticoagulants may differ systematically from those prescribed warfarin. While propensity score adjustment was employed in several studies [25], residual confounding cannot be excluded.

Additional evidence from registry-based studies aligns with these findings. The RAF-NOACs study (2017) reported lower major bleeding rates with direct oral anticoagulants versus Vitamin K Antagonists in acute ischemic stroke patients with atrial fibrillation, including reduced intracranial hemorrhage [26]. In older patients with atrial fibrillation and prior acute ischemic stroke, Xian et al. found direct oral anticoagulants linked to lower composite risks of death, readmission for stroke, or major bleeding, with specifically reduced intracranial hemorrhage [25]. Definitions of *recent acute ischemic stroke* varied across observational studies (from seven days to six months), limiting direct comparisons of absolute bleeding rates but not affecting the qualitative conclusion that direct oral anticoagulants are safer than Vitamin K Antagonists across the subacute to chronic post-stroke period [4,25,26].

Network meta-analyses suggest a hierarchy of bleeding risk among individual direct oral anticoagulant agents. Apixaban is consistently associated with the lowest risk of major bleeding and intracranial hemorrhage, followed by edoxaban and dabigatran (which have intermediate profiles), while rivaroxaban is associated with higher gastrointestinal bleeding risk [27-29]. Quantifying these differences, the network meta-analysis by Lopez-Lopez et al. reported absolute rates of major bleeding per 100 patient-years of approximately 2.1 for apixaban, 2.5 for edoxaban, 2.7 for dabigatran 150 mg, 3.0 for rivaroxaban, and 3.1 for warfarin [27]. For intracranial hemorrhage, absolute rates per 100 patient-years were 0.4 for apixaban, 0.5 for edoxaban, 0.3 for dabigatran 150 mg, 0.7 for rivaroxaban, and 0.8 for warfarin [27].

The safety rankings cited above reflect standard-dose regimens for each agent (apixaban 5 mg twice daily, dabigatran 150 mg twice daily, rivaroxaban 20 mg once daily, edoxaban 60 mg once daily). Dose-adjusted regimens - apixaban 2.5 mg twice daily (for patients with ≥2 of: age ≥80 years, weight ≤60 kg, serum creatinine ≥1.5 mg/dL), dabigatran 110 mg twice daily (for age ≥80 years or high bleeding risk), and edoxaban 30 mg once daily (for CrCl 15-50 mL/minute, weight ≤60 kg, or potent P-gp inhibitors) - show lower bleeding rates with potentially reduced efficacy [30,31]. Importantly, sensitivity analyses confirm that the relative safety rankings among agents remain consistent across dose-adjusted regimens [27].

Observational data suggesting lower intracranial hemorrhage recurrence with apixaban versus rivaroxaban in patients with breakthrough stroke are hypothesis-generating and require confirmation in prospective trials [32]. As noted in previous network meta-analyses, indirect comparisons and observational findings should not be interpreted as definitive evidence for switching agents without individualized assessment [33].

These safety hierarchies derived from general atrial fibrillation populations may not directly apply to the hyperacute post-stroke period, where blood-brain barrier disruption and cerebral autoregulation dysfunction create unique bleeding risks [34]. No head-to-head direct oral anticoagulant comparisons have been performed specifically in the first 7-14 days post-stroke, and most patients in the timing trials received apixaban, leaving other agents less studied [35]. Therefore, the observed differences in gastrointestinal bleeding and intracranial hemorrhage in chronic atrial fibrillation populations may not translate proportionally to the acute post-stroke setting.

This limitation underscores the need for a practical framework that synthesizes available evidence - a concept we operationalize as the *Safe Zone*.

Definition and basis of the *Safe Zone*

The *Safe Zone* describes the clinical and radiological window (typically 0-4 days) wherein early direct oral anticoagulant initiation is supported by randomized evidence. This term synthesizes the inclusion criteria and safety endpoints established by the ELAN, OPTIMAS, and TIMING trials, as well as their pooled analysis in the CATALYST meta-analysis [8-11].

The basis for the *Safe Zone* is twofold. First, statistical safety: the CATALYST meta-analysis established an exceptionally low absolute risk of symptomatic intracranial hemorrhage of 0.4% in the early initiation group, statistically equivalent to delayed initiation [11]. Second, physiological integrity: the zone is bounded by radiological markers (e.g., infarct volume ≤1/3 middle cerebral artery territory) and the absence of parenchymal hemorrhagic transformation (PH1 or PH2). The presence of only petechial changes (HI1/HI2) or no hemorrhage serves as a validated proxy for preserved blood-brain barrier integrity, allowing for early anticoagulation [36,37].

By identifying the *Safe Zone*, clinicians can move from a posture of *justified risk* to one of *evidence-based standard* for the majority of trial-concordant patients.

Clinical assessment for early anticoagulation

The practical question clinicians ask - Is this specific patient safe for early direct oral anticoagulant initiation (less than four days)? - is answered affirmatively for the majority of patients who match established trial profiles [11]. Safety is contingent on excluding high-risk features, particularly severe hemorrhagic transformation. Determining eligibility involves a comprehensive review of inclusion and exclusion criteria harmonized from the component trials of the CATALYST meta-analysis (Table 1).

**Table 1:** Clinical decision guide: risk stratification for early direct oral anticoagulant initiation. PH2, parenchymal hematoma type 2; PH1, parenchymal hematoma type 1; CrCl, creatinine clearance; SmPC, summary of product characteristics; Hb, hemoglobin; INR, international normalized ratio; MCA, middle cerebral artery; VKA, Vitamin K Antagonist; DAPT, dual antiplatelet therapy; mRS, modified Rankin scale; BBB, blood-brain barrier

Risk tier/phenotype	Trial representation (ELAN, OPTIMAS, TIMING)	Clinical guidance
Level 1 Red Flags: Absolute Contraindications		
Parenchymal Hematoma (PH2)	Universally Excluded. Patients with mass-effect hemorrhage (>30% of infarct) were not randomized.	Stop. Expert consensus suggests holding anticoagulation (often for ≥4 weeks) and repeating imaging to ensure stability and resolution of mass effect.
Mechanical Heart Valves/Mitral Stenosis	Universally Excluded. Trials were strictly limited to non-valvular atrial fibrillation.	Switch. Direct oral anticoagulants are contraindicated; Vitamin K Antagonists are mandated regardless of stroke timing.
End-Stage Renal Disease	Excluded. Trials utilized strict CrCl cutoffs (<15 or <30 mL/minute) or deferred to SmPC labels.	Consult Pharmacy. Dosing safety takes precedence; direct oral anticoagulants are generally avoided or used with extreme caution in end-stage renal disease, depending on local labeling.
Active Bleeding/Coagulopathy	Excluded: Serious bleeding <6 months; Platelets <100,000/mm³; Hb <10 g/dL; or INR ≥1.7.	Stop. Stabilize underlying bleeding risk, anemia, or thrombocytopenia before initiating anticoagulation.
High-Risk Vasculopathy	Excluded: Suspected Endocarditis, Aortic Dissection, or Cerebral Vasculitis.	Stop. Management of the primary underlying pathology takes priority over atrial fibrillation-related timing.
Refractory Hypertension	Controlled in Trials. Protocols required stabilization below 180/105 mmHg before enrollment or initiation.	Stabilize. Uncontrolled hypertension is a primary driver of blood-brain barrier disruption; stabilize blood pressure before starting anticoagulation.
Level 2 Red Flags: High-Risk "Caution" Scenarios		
Massive Infarction (>1/3 MCA)	Underrepresented. ELAN excluded "extraordinarily large" infarcts; OPTIMAS included only ~7%.	Protocol-Based Delay. For large infarcts, utilize initiation at Day 6-14 only after Radiological Safety Clearance confirms tissue stability.
Parenchymal Hematoma (PH1)	Weak Evidence. Often, a criterion for exclusion or delay in trial protocols.	Individualize. Consider initiation at Day 6-7 once clinical and radiological stability (no progression to PH2) is confirmed via re-imaging.
Pre-existing Anticoagulation	Excluded if on therapeutic VKA (INR ≥1.7) or direct oral anticoagulant at admission.	Stabilize. Delay initiation until the drug effect has cleared or the INR has normalized below the therapeutic threshold.
Chronic Dual Antiplatelet Therapy	Excluded if DAPT was required throughout the study period.	Consult Cardiology. Triple therapy (direct oral anticoagulant plus DAPT) carries a prohibitive bleeding risk; consider a "de-escalation" strategy.
Extreme Frailty (mRS >3)	Underrepresented. High pre-stroke disability and advanced age often led to later initiation in registries.	Assess Absolute Risk. Balance the benefit of preventing recurrence against the risk of falls and polypharmacy complications.
Pathological BBB Leakage	Mechanistic Risk. Associated with post-thrombectomy contrast extravasation and hemorrhagic transformation risk.	Monitor for hemorrhagic transformation. Significant contrast staining may justify a discretionary repeat scan 24-48 hours post-procedure before initiation.

Beyond the Red Flags in Table 1, the following criteria apply to early initiation (≤4 days). Common inclusion criteria include confirmed acute ischemic stroke via clinical and imaging assessment, documented atrial fibrillation, age ≥18 years, and eligibility for a licensed direct oral anticoagulant without absolute label contraindications [8-10]. Patients typically require CrCl >15 mL/minute (or >30 mL/minute for dabigatran). Mild hemorrhagic transformation (HI1/HI2) is generally permitted if the patient is stable and shows no clinical worsening contraindications [8-10,38].

Additional medical exclusions from the landmark trials include severe renal impairment or dialysis, significant liver dysfunction, known direct oral anticoagulant allergy, recent spontaneous intracerebral hemorrhage within the prior six months, and coagulopathy. Patients are also excluded if they have non-stroke pathology on imaging or if they are unable to participate in follow-up [8-10,38,39].

Conversely, several absolute contraindications necessitate exclusion from early initiation. Severe or moderate hemorrhagic transformation at baseline (specifically PH2) is a primary reason for exclusion, as is the presence of therapeutic anticoagulation at stroke onset [8,11]. Conditions mandating Vitamin K Antagonists - such as mechanical heart valves, moderate-to-severe mitral stenosis, or antiphospholipid syndrome - also disqualify patients from the ultra-early direct oral anticoagulant window [10,12].

Optimal timing within the early window

Once early direct oral anticoagulant initiation is deemed safe, the question remains: how early is optimal? The CATALYST individual participant data meta-analysis provides global support for initiation within four days or less but lacks granular day-by-day subgroups; medians across the pooled data suggest that days 2-3 are the most common points of initiation [11] . Statistical framing from this meta-analysis established an absolute risk of symptomatic intracranial hemorrhage of only 0.4% in the early group, demonstrating a robust *safety floor* for the 0-4 day window.

While median initiation times provide a useful summary, they obscure the distribution of actual start times. In CATALYST, the early group included patients starting as early as day 1 and as late as day 4 [11]. The safety data apply to the entire 0-4 day window, but the optimal timing within that window may differ based on infarct size, as detailed in Step 4.

Data for initiation within the first 24 hours are limited. In CATALYST, only 12% of patients in the early group started on Day 1, and safety estimates for this ultra-early window are, therefore, less precise [11]. For patients presenting within 24 hours of symptom onset, the protocol recommends waiting until Days 1-2 for minor strokes, effectively meaning 24-48 hours post-onset, rather than immediate initiation. This approach aligns with expert consensus that very early initiation should be reserved for patients with transient ischemic attack and no visible infarct [40].

The specific day-by-day recommendations in this protocol (Days 1-2 for minor, Days 2-3 for moderate, Day 4 for major) are extrapolated from trial protocols and median initiation times rather than derived from direct randomization to these discrete timepoints. No trial has randomized patients to Day 1 versus Day 2 versus Day 3. Within the 0-4 day window established by CATALYST, the precise optimal day for an individual patient is determined by integrating infarct size, clinical stability, and hemorrhagic status, as operationalized in Step 4. The 0-4 day *Safe Zone* should be interpreted as a target range, not a maximum threshold.

A critical finding in contemporary stroke neurology is that radiological infarct size correlates more accurately with the risk of hemorrhagic transformation than clinical deficit scores, such as the National Institutes of Health Stroke Scale [36,41]. This creates a *translational gap* where relying solely on clinical severity can lead to either unnecessary delays or hazardous early starts. As demonstrated in Table 2, the use of imaging volume is superior to clinical data for identifying these mismatches and optimizing timing.

To ensure clinical reproducibility, we have adopted the specific radiological definitions utilized in the ELAN trial to guide timing. These definitions form the basis for the timing recommendations in Step 4.

**Table 2:** Clinical-imaging dissociation and its impact on direct oral anticoagulant timing. NIHSS, National Institutes of Health Stroke Scale; MCA, middle cerebral artery; PCA, posterior cerebral artery; PH2, parenchymal hematoma type 2; PH1, parenchymal hematoma type 1; MT, mechanical thrombectomy; IVT, intravenous thrombolysis

Stroke phenotype	Clinical severity (NIHSS)	Imaging volume (ELAN criteria)	Risk of clinical-imaging mismatch	Recommended timing strategy
*Small-but-Severe* (Eloquent stroke)	High (e.g., >15): Dense hemiplegia or global aphasia	Minor (≤1.5 cm): Small focal lesion (e.g., lacunar or brainstem)	Over-estimation of Risk: NIHSS suggests a major stroke, but low tissue volume carries a very low absolute risk of hemorrhagic transformation.	Initiate Early (Days 1-2): Prioritize the low radiological volume. Delaying based on NIHSS increases recurrence risk without a safety benefit.
*Large-but-Silent *(Non-dominant stroke)	Low (e.g., <8): Mild neglect or subtle weakness	Major (Full Territory): Infarct involving the entire MCA or PCA territory	Under-estimation of Risk: Low NIHSS suggests a minor stroke, but massive tissue loss correlates with severe blood-brain barrier disruption.	Delay Initiation (Days 6-7): Prioritize the high radiological volume. Early initiation based on low NIHSS risks symptomatic PH2.
Reperfused stroke (Post-MT/IVT)	Variable: Often demonstrates rapid recovery	Variable: May show *ischemic stunning* or contrast extravasation	Fluctuating Risk: Clinical improvement may mask underlying vascular fragility and blood-brain barrier *leakage*.	Stability-Based: Re-image at 24 hours. If no PH1/PH2 is present, follow volume-based timing (Step 4).

Minor or Small Stroke

Transient ischemic attack or small infarct ≤1.5 cm on non-contrast CT or MRI (typically lacunar or small cortical lesions). The 1.5 cm threshold was derived from CT measurements in the ELAN trial. MRI, with higher sensitivity for small ischemic lesions, may identify additional minor infarcts not visible on CT, but the threshold applies to the largest dimension measured on any modality, consistent with the ELAN protocol's acceptance of both imaging modalities for stroke severity classification [8]. In this cohort, ultra-early initiation demonstrated an exceptionally low symptomatic intracranial hemorrhage rate of 0.2% [8,20].

Moderate Stroke

Infarct in the distribution of a single superficial cortical branch of the MCA, ACA, or PCA, or a cerebellar or brainstem infarct >1.5 cm. For moderate and major strokes, the territorial definitions are modality-independent, as they are based on vascular distribution rather than absolute measurements. The ELAN criteria apply to both anterior and posterior circulation infarcts, and the same timing logic applies regardless of vascular territory [8]. This aligns with median timing observed in CATALYST and TIMING [8,10,11].

Major or Severe Stroke

Large territory infarct involving the entire distribution of the MCA, ACA, or PCA (or large portions of the cerebellum or brainstem). These patients require consideration of the higher intrinsic risk of blood-brain barrier disruption and parenchymal hematoma [8,41].

Subgroup analysis confirms that the primary efficacy of early initiation - notably a 34% reduction in the odds of recurrent stroke - is maintained across high-risk groups. Specifically, reperfusion therapy, CKD (if CrCl >15 mL/minute), and advanced age (over 75 years) do not necessitate systematic delay within the four-day window if the patient remains clinically stable [8,9,11].

Neuroimaging protocols

Once timing is determined, the role of imaging - both before and after initiation - must be considered. Major randomized trials do not mandate routine repeat neuroimaging in asymptomatic patients, as symptomatic intracranial hemorrhage rates are consistently low and asymptomatic hemorrhagic transformation rarely alters clinical management [8-10,39]. Regarding pre-initiation imaging, no routine repeat imaging is necessary if baseline CT or MRI excludes disqualifying hemorrhagic transformation (specifically PH2) and the patient remains clinically stable [11,42].

Inter-reader variability in detecting mild hemorrhagic transformation, particularly the distinction between HI1/HI2 and more subtle findings, is well-recognized in the literature [43,44]. Studies have shown only fair to moderate agreement among physicians in identifying petechial hemorrhage on non-contrast CT, with agreement improving substantially only for large parenchymal hematomas (PH2). This evidence base justifies the *Conservative Discordance* rule introduced in Step 2.

However, repeat imaging remains indicated in three specific clinical scenarios:

(1)* Clinical deterioration*: Any new or worsening neurological deficit (e.g., increase in NIHSS score) warrants immediate non-contrast CT or MRI to rule out symptomatic intracranial hemorrhage or significant expansion of baseline hemorrhage [45].

(2) *Radiological safety clearance*: Required for patients with major territory infarcts or PH1 where initiation is planned for Days 6-7; a repeat scan on the day of planned start is required to ensure tissue stability and absence of late-evolving parenchymal hematoma [8,9].

(3)* Discretionary high-risk monitoring*: May be justified for baseline high-risk features, such as very large infarct volume or initial petechial hemorrhagic transformation (HI1/HI2), warranting a discretionary scan 24-48 hours before starting anticoagulation to verify absence of progression [8,9].

Reperfusion therapy (intravenous thrombolysis (IVT) or mechanical thrombectomy (MT)) may increase the risk of hemorrhagic transformation independent of infarct size by inducing reperfusion injury and blood-brain barrier disruption [46]. In the ELAN trial, patients receiving reperfusion therapy were included and did not show excess bleeding with early initiation, with prior reperfusion not modifying the treatment effect [47].

For patients with large infarcts who undergo successful reperfusion, a discretionary repeat scan at 24 hours is recommended to exclude reperfusion-related hemorrhage before proceeding with infarct size-based timing. This interval is consistent with established guidelines for follow-up imaging after both thrombolysis and mechanical thrombectomy [48]. The 24-hour window allows for resolution of contrast extravasation, which may otherwise be mistaken for true hemorrhage, while persistent hyperdensities at this time point have high specificity for parenchymal hematoma [48].

Following initiation of anticoagulation, there is no evidence-based recommendation for routine screening for asymptomatic hemorrhagic transformation [11,42,49]. Data from CATALYST confirm that asymptomatic hemorrhagic transformation rarely progresses to symptomatic intracranial hemorrhage when utilizing direct oral anticoagulants, provided initial risk stratification was accurate [11]. Subsequent imaging should be reserved strictly for patients demonstrating new or worsening symptoms [45].

Perfusion imaging (CTP or MRP) and susceptibility-weighted imaging may detect additional findings such as tissue at risk or occult microhemorrhage [50,51], but their role in direct oral anticoagulant timing decisions has not been prospectively validated and they were not required in the component trials.

For centers without reliable access to repeat imaging, an alternative approach is required. In resource-limited settings where radiological safety clearance is unavailable, a conservative 14-day delay is recommended for high-risk phenotypes (major territory infarcts or PH1). This represents the upper bound of the OPTIMAS delayed arm (7-14 days) [9] and aligns with the historical *1-3-6-12 day* rule upper bound [52]. This default acknowledges the trade-off: delaying beyond Day 7 may increase the risk of recurrent ischemic stroke [53] but prioritizes the prevention of hematoma expansion in the absence of radiological data.

Synthesized step-by-step protocol

**Figure 1:**
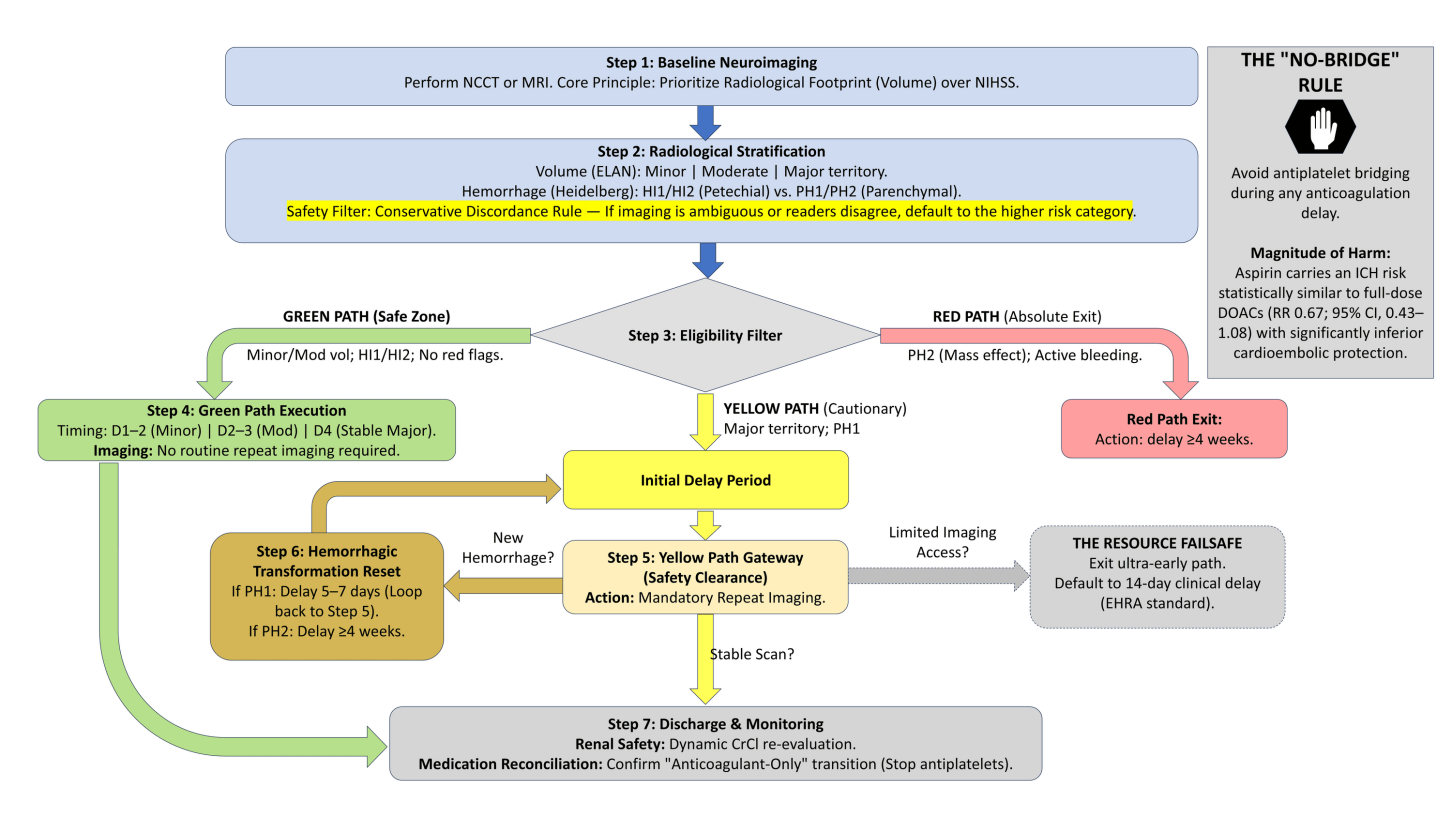
Unified seven-step protocol and imaging gateway for direct oral anticoagulant (DOAC) initiation. A structured clinical decision-making framework for the initiation of DOACs following atrial fibrillation (AF)-related acute ischemic stroke (AIS). The protocol prioritizes the radiological footprint (infarct volume and hemorrhagic status) over clinical National Institutes of Health Stroke Scale (NIHSS) scores to enhance safety and precision. Steps 1-2 (Diagnostic Foundation): Initial stratification utilizes the ELAN radiological criteria for volume and the Heidelberg Classification for hemorrhagic transformation (HT). The Conservative Discordance Rule mandates that any ambiguity in imaging interpretation defaults to a higher-risk category to prevent premature initiation. Step 3 (Eligibility Filter): Patients are triaged into three pathways: the Green Path (trial-concordant Safe Zone), the Yellow Path (cautionary or high-risk phenotypes), and the Red Path (absolute exclusion for parenchymal hematoma type 2 (PH2) or mechanical valves). Steps 4-6 (Execution and Failsafes): The Green Path supports *ultra-early* initiation (Days 1-4) without routine repeat imaging. The Yellow Path requires mandatory Radiological Safety Clearance (repeat scan) at Step 5. If imaging reveals new HT, the clock is reset (Step 6). The Resource Failsafe: in settings with limited imaging access, the protocol defaults to a conservative 14-day clinical delay, aligning with the European Heart Rhythm Association (EHRA) 1-3-6-12 standard to prioritize safety. Core Evidence Sidebar: Highlights the categorical avoidance of antiplatelet bridging. Recent meta-analytic data indicate that aspirin carries an intracranial hemorrhage (ICH) risk statistically similar to full-dose DOACs (risk ratio (RR) 0.67; 95% confidence interval (CI), 0.43-1.08) while providing significantly inferior protection against cardioembolic recurrence. Step 7 (Discharge and Monitoring): Focuses on renal monitoring (CrCl) and the anticoagulant-only transition to minimize long-term bleeding complications. Image credit: Deb Mojumder.

A synthesized step-by-step protocol is shown in Figure 1.

Step 1: Confirm Diagnosis and Baseline Imaging

Perform urgent neuroimaging-ideally non-contrast CT, or MRI where available-to confirm acute ischemic stroke and exclude primary intracerebral hemorrhage or stroke mimics. Document NIHSS score for clinical baseline, but prioritize radiological footprint and hemorrhagic transformation status for anticoagulation timing. This imaging-centric approach is critical because clinical scores can be misleading; a low NIHSS may mask significant *silent* tissue injury, while a high NIHSS in an eloquent area may over-represent biological risk of blood-brain barrier disruption [8,37,45].

If baseline imaging is suboptimal (e.g., motion artifact, incomplete coverage) or delayed beyond 24 hours post-admission, default to a conservative approach guided by clinical assessment. For patients with suspected large infarct or high clinical severity (e.g., NIHSS >10), obtain a repeat scan before proceeding. If clinical deterioration occurs while awaiting imaging, immediate repeat imaging is mandatory [45].

Step 2: Stratify Stroke Severity and Hemorrhagic Status

Using baseline images, stratify stroke according to ELAN radiological criteria (detailed above) and Heidelberg hemorrhagic transformation classification. This step serves as the definitive proxy for assessing blood-brain barrier structural integrity.

Minor stroke: Transient ischemic attack or small infarct ≤1.5 cm in any territory (anterior or posterior circulation)

Moderate stroke: Infarct involving a single superficial cortical branch of the MCA, ACA, or PCA; internal border zone infarct; or cerebellar or brainstem infarct >1.5 cm not involving entire territory

Major stroke: Large territory infarct involving entire distribution of MCA, ACA, or PCA; infarcts spanning multiple territories; or brainstem infarcts with significant mass effect

Hemorrhagic status requires grading using Heidelberg criteria [36]:

HI1 or HI2 (petechial): Small, confluent petechiae without space-occupying effect-low risk

PH1 (moderate): Focal blood clot involving ≤30% of infarct area with negligible mass effect

PH2 (severe): Dense blood clot involving >30% of infarct area with significant mass effect

To mitigate inter-reader variability in identifying subtle petechial hemorrhage (HI1/HI2) - a challenge well-documented in the literature [43,44] - this protocol adopts a principle of *Conservative Discordance*. In cases where interpretation of baseline imaging is ambiguous or where discrepancies exist between clinical and radiological readings, clinicians must default to the more conservative timing tier (e.g., Moderate instead of Minor) to ensure maximum blood-brain barrier stabilization.

Step 3: Determine Eligibility via the "Safe Zone" Filter

Run radiological and clinical labels from Step 2 through this gate to determine therapeutic track. Entry into the *Safe Zone *- where early initiation (0-4 days) is evidence-based standard - requires passing a *double-lock *check of trial-concordant inclusion and absolute absence of Red Flags, as defined above and in Table 1.

Safe Zone inclusion: Confirmed acute ischemic stroke with non-valvular atrial fibrillation, clinical stability, and preserved renal function (CrCl >15 mL/minute; >30 mL/minute for dabigatran)

Level 1 Red Flags (Absolute - Exit Protocol)

Valvular pathology: mechanical heart valves or moderate-to-severe rheumatic mitral stenosis

Imaging findings: PH2 (dense blood clot >30% of infarct area with mass effect)

Other: reversible causes of atrial fibrillation (e.g., thyrotoxicosis) or active major bleeding

Action for Level 1: Stop or switch-transition to Vitamin K Antagonists for valvular atrial fibrillation or delay ≥4 weeks for PH2 until mass effect resolves [8,10].

Level 2 Red Flags (Cautionary - Divert to Step 5)

Anatomy/hemorrhage: massive territory infarcts (Major) or PH1 (focal blood clot ≤30% of area)

Hemodynamics: refractory hypertension (persistent BP >180/105 mmHg)

Baseline status: therapeutic-dose anticoagulation at stroke onset (VKA with INR ≥1.7 or elevated direct oral anticoagulant levels)

Action for Level 2: Delay initiation (typically 5-14 days) and mandate *Radiological Safety Clearance* scan in Step 5 [8,11].

Step 4: Determine Standard Timing Within the "Safe Zone"

For patients in the Safe Zone (Minor or Moderate volume, no PH1 or PH2), initiation timing is guided strictly by radiological footprint identified in Step Two, using the ELAN definitions detailed above. This imaging-first approach is validated as safe regardless of age, sex, prior reperfusion therapy (IVT or MT), or mild-to-moderate renal impairment [9,11].

Minor strokes (TIA or infarct ≤1.5 cm): Initiate direct oral anticoagulant on Day 1 or 2 (≤48 hours)

Moderate strokes (single superficial branch): Initiate direct oral anticoagulant on Day 2 or 3

Major strokes (stable, non-massive-large infarcts with no mass effect or PH1/PH2): Initiate on Day 4

For major strokes, the CATALYST meta-analysis provides a critical evidence-based override to ELAN's Day 6-7 recommendation. In the CATALYST severe stroke subgroup (NIHSS ≥16), initiation within <4 days resulted in a primary outcome rate of 1.5% vs 2.7% [11]. Meta-analytic data from 5,470 participants confirms that initiation within <4 days provides a 34% reduction in recurrent ischemic stroke with an absolute symptomatic intracranial hemorrhage risk of 0.4% [11]. This suggests that for patients with major strokes who are clinically stable and lack Red Flag imaging, Day 4 serves as a validated safety floor, merging ELAN's imaging precision with CATALYST's statistical power. For these "Green Light" patients, routine repeat imaging is not required unless clinical deterioration occurs.

Step 5: Address Repeat Imaging and "Safety Clearance"

Neuroimaging serves as the final "Safety Gateway" before initiation. In the post-CATALYST era, repeat imaging has shifted from routine requirement to targeted safety check for high-risk phenotypes where blood-brain barrier integrity is in question, as detailed above.

Safe Zone patients (minor/moderate stroke, no hemorrhagic transformation): Routine repeat neuroimaging is not recommended before starting anticoagulation unless clinical status changes. CATALYST established an exceptionally low 0.4% absolute risk of symptomatic intracranial hemorrhage in early initiation, justifying this streamlined approach [11].

High-risk phenotypes (PH1 or major territory infarcts): Mandatory repeat scan on Days 6-7 for definitive *Radiological Safety Clearance* to ensure hematoma or infarct tissue is stable before anticoagulation. While ELAN post-hoc analysis confirms low symptomatic intracranial hemorrhage risk with early treatment, trend toward poorer 90-day functional outcomes (mRS 3-6) justifies waiting until Days 6-7 to confirm hematoma stability [54]. Balancing this caution, observational data indicate that recurrent ischemic stroke risk with delayed initiation rises across the hemorrhagic transformation spectrum: from 3.7% (no hemorrhagic transformation), to 6.3% (hemorrhagic infarction), to 15.4% (parenchymal hematoma) [53]. Once stability confirmed on Days 6-7 scan, initiate promptly to mitigate escalating embolic risk.

PH2 (parenchymal hematoma type 2): Excluded from ultra-early window entirely. Repeat imaging deferred to four-week mark, as early initiation in this cohort is associated with significantly higher risk of poor functional outcomes [54]. Lacking dedicated post-stroke data, timing extrapolated from spontaneous intracerebral hemorrhage-meta-analytic data (pooled mean 31 days) and expert consensus support a 4-6 week window for safe resumption [55,56].

Clinical deterioration: Any acute neurological decline or significant increase in NIHSS mandates immediate repeat imaging to assess for intracranial hemorrhage or infarct expansion, regardless of initial zone status [45].

Post-initiation monitoring: Routine screening for asymptomatic hemorrhagic transformation after first direct oral anticoagulant dose is not indicated. Late-occurring petechial changes (HI1/HI2) are common and do not warrant therapy cessation [49], nor do they correlate with worse clinical outcomes [42].

Step 6: Utilize Hemorrhagic Transformation as a Timing Reset

If hemorrhagic transformation is identified at any stage, it becomes the definitive determinant for the initiation clock, overriding standard clinical categories. Management is stratified by Heidelberg Classification to balance hematoma expansion risk against recurrent ischemia threat.

Petechial hemorrhagic transformation (HI1/HI2 - Safe Zone): These changes represent minor diapedesis across semi-permeable blood-brain barrier rather than structural vascular rupture. CATALYST confirms early initiation in presence of HI1/HI2 does not increase absolute symptomatic intracranial hemorrhage risk (0.4%) [11]. Patients with HI1/HI2 remain in Safe Zone; timing dictated strictly by radiological infarct size per Step 4 [8,39].

Parenchymal hematoma (PH1 and PH2): Distinction based on Heidelberg Bleeding Classification, validated as predictor of functional outcome and mortality [36]. Pooled outcome data indicate PH1 carries 30-day mortality of approximately 15% to 20%, compared to 50% to 60% for PH2 [57].

PH1 (Level 2 Red Flag): PH1 represents distinct focal blood clot involving ≤30% of infarct area with negligible mass effect, signifying structural vascular wall breach. Evidence base for PH1 timing is limited.

ELAN trial protocol excluded PH1, but central re-adjudication identified 34 participants with PH1 (1.7% of cohort) enrolled [54]. In this small subgroup, early initiation was associated with trend toward worse 90-day functional outcomes (adjusted risk difference 25.1%), though confidence intervals wide and event rates low. Observational data suggest initiating direct oral anticoagulants at median seven days in patients with parenchymal hematoma appears safe, with no worsening of hemorrhage after initiation [53].

Synthesizing this evidence-concerning signal from the ELAN subgroup, observational data showing safe initiation at a median of seven days, and the universal exclusion of PH1 patients from early randomized trial arms, we recommend a cautious delay of five to seven days (i.e., scan at Day 6 at the earliest) for PH1. This delay must be followed by mandatory Radiological Safety Clearance scan (Step 5) before therapy begins. This recommendation represents a consensus-based approach, as randomized data specifically for PH1 are lacking.

If repeat imaging after the initial delay of five to seven days shows persistent PH1 without expansion, anticoagulation may be initiated with close clinical monitoring. If PH1 progresses to PH2, a further delay of at least four weeks with repeat imaging is required before reconsidering anticoagulation.

The optimal reassessment interval if PH1 persists unchanged without progression has not been established. The natural history of PH1 expansion is not well characterized, and the introduction of anticoagulation could theoretically alter this trajectory by preventing thrombus stabilization or promoting hematoma enlargement. In such cases of diagnostic uncertainty, clinical judgment and multidisciplinary input, including stroke neurology and neurosurgery, are recommended.

PH2 (Level 1 Red Flag): PH2 involves dense clot involving >30% of infarct area with significant mass effect, representing total blood-brain barrier failure. This is absolute contraindication to early anticoagulation. Initiation must be delayed for at least four weeks, with follow-up scan mandated to confirm resolution of mass effect and clot retraction before starting direct oral anticoagulant [7,8].

When hemorrhagic transformation is identified, clinicians may consider its location, though evidence for differential management based on location in hemorrhagic transformation is lacking. In spontaneous intracerebral hemorrhage, deep hemorrhages involving the basal ganglia or thalamus carry a high risk of functional deterioration with hematoma expansion due to the eloquence of these brain regions [58,59]. The proximity to critical motor and sensory pathways, such as the posterior limb of the internal capsule, means that even small expansions can have catastrophic functional consequences [60]. However, hemorrhagic transformation of an ischemic infarct is biologically distinct from spontaneous intracerebral hemorrhage, and data from the spontaneous intracerebral hemorrhage population cannot be directly extrapolated to hemorrhagic transformation. No studies have established whether deep versus lobar location should independently influence direct oral anticoagulant timing in the setting of hemorrhagic transformation. Therefore, the protocol's severity-based framework (HI1/HI2, PH1, PH2) remains the primary guide. Location should be considered as an additional factor in individualized clinical judgment, recognizing that expansion in eloquent areas-if it were to occur-could be devastating, rather than as an evidence-based determinant of timing.

The *No-Bridge *Rule applies across all hemorrhagic transformation categories: antiplatelet bridging must be avoided during any anticoagulation delay. Evidence confirms bridging increases risk of symptomatic hematoma expansion without providing superior protection against recurrent stroke. Notably, with relative risk of 0.67 for apixaban versus aspirin, use of antiplatelets as a *safe* bridge lacks statistical support [7].

Step 7: Monitor and Dynamic Follow-Up

The final step ensures safety established in acute phase is maintained throughout critical 90-day window and beyond. Clinical monitoring focuses on triad of recurrent stroke, symptomatic intracranial hemorrhage, and systemic embolism [11].

Dynamic renal monitoring: Direct oral anticoagulant safety is contingent on accurate dosing. Because renal function can be labile in post-stroke period (due to contrast exposure or acute illness), CrCl should be re-evaluated before discharge and at first outpatient follow-up. Dosages must be adjusted dynamically for changes in age, weight, or renal function per manufacturer guidelines [9,38]. For patients with borderline renal function (CrCl 15-30 mL/minute) or fluctuating creatinine, repeat measurement within 24-48 hours is recommended before initiating or adjusting direct oral anticoagulant therapy [61]. Dose selection should be based on most recent stable value, with early outpatient follow-up and repeat renal function testing warranted.

Agent selection by renal clearance: As detailed above, direct oral anticoagulants have varying renal clearance profiles. Dabigatran (80% renal) requires caution with CrCl <30 mL/minute; rivaroxaban (33%-35% renal) contraindicated if CrCl <15 mL/minute; apixaban (25%-27% renal) can be used with dose adjustment down to CrCl ≥15 mL/minute; edoxaban (50% renal) not recommended if CrCl >95 mL/minute due to reduced efficacy [62,63].

The CrCl thresholds cited reflect the inclusion criteria of the landmark timing trials. The ELAN trial required CrCl ≥30 mL/minute for dabigatran and allowed other direct oral anticoagulants at physician discretion per local labeling; the OPTIMAS trial required CrCl ≥15 mL/minute for all direct oral anticoagulants [8,9]. For consistency with the imaging-based framework, this protocol aligns with ELAN's approach: dabigatran is reserved for patients with CrCl ≥30 mL/minute, while apixaban, rivaroxaban, and edoxaban may be used with appropriate dose adjustment down to CrCl ≥15 mL/minute, in accordance with regulatory labeling and local prescribing information.

OPTIMAS CKD sub-study confirmed early initiation safe across all CKD stages with appropriate dose adjustment, though data for end-stage renal disease (estimated glomerular filtration rate (eGFR) <15) remain limited [38].

Imaging surveillance: Routine follow-up neuroimaging is not indicated in absence of new neurological symptoms. Late-occurring, asymptomatic petechial changes are common and do not necessitate therapy cessation [49].

Medication reconciliation (*Anticoagulant-Only *transition): Unless specific concurrent indication exists (e.g., recent coronary stenting), antiplatelet therapy should typically be discontinued once direct oral anticoagulant is initiated. Maintaining unnecessary *double-coverage* is primary driver of late-stage bleeding complications and is not supported by current atrial fibrillation-stroke evidence [7,12].

Transitions of care: Formal handover to primary care or cardiology is required to ensure long-term adherence. Include clear documentation of specific direct oral anticoagulant used, *Safe Zone* timing rationale, and required schedule for future renal and hematological monitoring. Table 3 summarizes the essential steps.

**Table 3:** Summary of seven-step protocol for direct oral anticoagulant initiation after atrial fibrillation-related stroke. CT, computed tomography; MRI, magnetic resonance imaging; NIHSS, National Institutes of Health Stroke Scale; CrCl, creatinine clearance; HI1, hemorrhagic infarction type 1; HI2, hemorrhagic infarction type 2; PH1, parenchymal hematoma type 1; PH2, parenchymal hematoma type 2

Step	Action	Key considerations	Timing/Threshold
Step 1	Confirm diagnosis and baseline imaging	Urgent non-contrast CT/MRI; document NIHSS but prioritize radiological footprint	If suboptimal imaging, repeat before proceeding
Step 2	Stratify severity and hemorrhagic transformation status	ELAN criteria (minor ≤1.5 cm, moderate single branch, major full territory); Heidelberg hemorrhagic transformation classification	"Conservative Discordance" rule for ambiguity
Step 3	Safe zone eligibility	Non-valvular atrial fibrillation, clinical stability, CrCl >15 (>30 for dabigatran)	Level 1 Red Flags: Exit protocol; Level 2: Divert to Step 5
Step 4	Standard timing	Minor: Day 1-2; Moderate: Day 2-3; Major (stable): Day 4	CATALYST meta-analysis supports Day 4 for major strokes (vs. ELAN Days 6-7)
Step 5	Safety clearance imaging	No routine repeat for Safe Zone; mandatory Days 6-7 for PH1/major infarct; PH2 delay ≥4 weeks	Resource-limited: default 14-day delay
Step 6	Hemorrhagic transformation as timing reset	HI1/HI2: follow Step 4; PH1: delay five to seven days with repeat imaging; PH2: delay ≥4 weeks	*No-Bridge *Rule: avoid antiplatelets
Step 7	Monitor and follow up	Dynamic renal monitoring; agent selection by CrCl; anticoagulant-only transition	Re-check CrCl before discharge and at first follow-up

Long-term outcomes and the temporal advantage

At 90-day follow-up, absolute reduction in primary composite events (recurrent stroke, systemic embolism, or major bleeding) for early initiation group was numerically maintained but did not retain sharp statistical significance observed in ultra-acute phase [11]. This gradual dilution of effect is a recognized phenomenon in stroke trials, as the 90-day window introduces competing risks unrelated to the initial timing decision, such as medication non-adherence, secondary comorbidities, and long-term hemodynamic variability [8,9].

The distinct clinical advantage of early direct oral anticoagulant initiation is localized to the *High-Risk Acute Window *- specifically the first 14 days post-stroke [11,37]. During this period, the brain is in hyper-acute pro-thrombotic state where hazard ratio for recurrent embolism is at its zenith. By utilizing the *Safe Zone* (0-4 days) for initiation, clinicians effectively neutralize this early peak in ischemic risk without reciprocal increase in symptomatic hemorrhage-a benefit that remains primary justification for the *Post-CATALYST* paradigm shift [9,11]. In delayed initiation arms, recurrent ischemic events clustered in first 7-10 days post-stroke. In the ELAN trial, 80% of recurrent events in the delayed arm occurred before anticoagulation was initiated [8].

Future directions

A significant limitation of the current CATALYST meta-analysis was the lack of centralized, core lab-adjudicated imaging data for the entire pooled cohort [11]. While the study established a population-level *safety floor*, reliance on site-reported radiological data limits ability to perform granular, voxel-based risk modeling. Consequently, a large-scale *Timing-Risk* sub-analysis is currently underway to evaluate baseline and follow-up brain images directly [11].

This future study aims to move beyond categorical *day-based* bins by exploring mathematical relationship between objective infarct volume, specific grade of hemorrhagic transformation, and direct oral anticoagulant initiation timing treated as continuous variable [11,37]. Such *precision medicine* approach will likely identify exact physiological tipping point where risk of blood-brain barrier failure outweighs benefit of early anticoagulation, potentially allowing further refinement of *Safe Zone* boundaries established in this review.

## Conclusions

The post-CATALYST era has fundamentally redefined the management of atrial fibrillation-related acute ischemic stroke, shifting the clinical imperative from anticoagulation delay to informed, early initiation. The synthesis of landmark trials (ELAN, OPTIMAS, TIMING) within the 2025 CATALYST meta-analysis establishes a clear *Safe Zone* for direct oral anticoagulant initiation within four days, a window that reduces recurrent events by 34% without increasing the 0.4% baseline risk of symptomatic hemorrhage. This review provides the operational roadmap for this transition, harmonizing population-level safety with imaging-based precision through a unified Seven-Step Protocol.

By anchoring decisions to the biological determinants of bleeding, namely radiological infarct volume and blood-brain barrier integrity, this framework replaces Vitamin K Antagonist-era conservatism with a structured, evidence-based pathway. Its core contributions translate high-level data into actionable decisions for the multidisciplinary stroke team: a tiered approach to hemorrhagic transformation that validates early initiation for HI1/HI2 while mandating delay for PH1 and PH2; the *Conservative Discordance* rule to mitigate imaging ambiguity; and a categorical prohibition of antiplatelet bridging, a practice now rendered obsolete by evidence.

However, the boundaries of this new era must be honestly delineated. For the majority of trial-concordant patients, this protocol offers a definitive path. Yet the evidence remains insufficient for populations systematically excluded from major trials, including those with severe strokes (NIHSS >20), extremely large infarcts (>1/2 MCA territory), or extensive parenchymal hematomas (PH2). For these patients, as well as those with complex multi-morbid conditions, the protocol's recommendations should be applied with extreme caution, often requiring multidisciplinary input and delayed initiation. Furthermore, the safety hierarchies of individual direct oral anticoagulants established in chronic atrial fibrillation cannot be assumed to apply perfectly to the hyperacute post-stroke period, where cerebral autoregulation is uniquely dysfunctional.

The recommendations in this protocol are derived from trial efficacy and safety data and do not incorporate cost-effectiveness or resource availability considerations. Further research is needed to evaluate the cost-effectiveness of imaging-based timing strategies and to optimize implementation in resource-limited settings.

The next horizon for the field, therefore, is one of refinement. Centralized, voxel-based imaging analyses are needed to move from categorical timing to continuous risk models, and rigorous implementation science is required to ensure these advances are equitably adopted across all practice settings. This protocol is intended to complement existing guideline authority by providing the day-by-day operational detail necessary for modern practice. The post-CATALYST era has provided the foundation; the work ahead lies in perfecting its application to achieve the best possible outcome for every patient.
